# Analysis of genes that are differentially expressed during the *Sclerotinia sclerotiorum*–*Phaseolus vulgaris* interaction

**DOI:** 10.3389/fmicb.2015.01162

**Published:** 2015-10-26

**Authors:** Marília B. Oliveira, Rosângela V. de Andrade, Maria F. Grossi-de-Sá, Silvana Petrofeza

**Affiliations:** ^1^Departamento de Bioquimica e Biologia Molecular, Instituto de Ciências Biológicas, Universidade Federal de GoiásGoiânia, Brazil; ^2^Programa de Pós-graduação em Ciências Genômicas e Biotecnologia, Universidade Católica de BrasíliaBrasília, Brazil; ^3^Embrapa Recursos Genéticos e Biotecnologia, Laboratório de Interação Molecular Planta-PragaBrasília, Brazil

**Keywords:** necrotrophic fungi, common bean, gene expression, *Sclerotinia sclerotiorum*, suppression subtractive hybridization, stress-induced transcripts

## Abstract

The fungus *Sclerotinia sclerotiorum* (Lib.) de Bary, one of the most important plant pathogens, causes white mold on a wide range of crops. Crop yield can be dramatically decreased due to this disease, depending on the plant cultivar and environmental conditions. In this study, a suppression subtractive hybridization cDNA library approach was used for the identification of pathogen and plant genes that were differentially expressed during infection of the susceptible cultivar BRS Pérola of *Phaseolus vulgaris* L. A total of 979 unigenes (430 contigs and 549 singletons) were obtained and classified according to their functional categories. The transcriptional profile of 11 fungal genes related to pathogenicity and virulence were evaluated by reverse transcription quantitative real-time polymerase chain reaction (RT-qPCR). Additionally, the temporal expression profile obtained by RT-qPCR was evaluated for the following categories of plant defense-related genes: pathogenesis-related genes (*PvPR1*, *PvPR2*, and *PvPR3*), phenylpropanoid pathway genes (*PvIsof*, *PvFPS1*, and *4CL*), and genes involved in defense and stress-related categories (*PvLox*, *PvHiprp*, *PvGST*, *PvPod*, and *PvDox*). Data obtained in this study provide a starting point for achieving a better understanding of the pathosystem *S. sclerotiorum*–*P. vulgaris.*

## Introduction

Common beans (*Phaseolus vulgaris* L.) are the world’s most important grain legume for direct human consumption (Broughton et al., 2003). However, bean production is affected by several diseases, such as white mold, which is one of the most important diseases that affect the quality and yield of crops on a global scale. The pathogen associated with white mold, *Sclerotinia sclerotiorum* (Lib.) de Bary, is a necrotrophic pathogen with worldwide distribution and is known to infect over 400 species of plants (Boland and Hall, 1994). The sclerotia of this pathogen are capable of surviving in the soil for several years and infect the majority of hosts indirectly, i.e., they germinate to produce apothecia, which release ascospores (Bolton et al., 2006). Because of its ability to infect economically important crops causing major losses, *S. sclerotiorum* has been the focus of many research programs. As a consequence, scientists have successfully annotated its genome (Amselem et al., 2011), investigated the fungus at the level of gene expression, and performed proteome-level studies (Yajima and Kav, 2006).

Previous studies on the pathogenicity of *S. sclerotiorum* mainly focused on the secretion of oxalic acid (Lumsden, 1979; Godoy et al., 1990) and hydrolytic enzymes (Marciano et al., 1983; Cotton et al., 2003), which act in concert to macerate plant tissues and generate necrosis. Lytic enzymes, such as cellulases, hemicellulases, pectinases, and proteases, sequentially secreted by the fungus facilitate penetration, colonization, and maceration and also generate an important source of nutrients (Hegedus and Rimmer, 2005; Bolton et al., 2006). However, oxalic acid is likely to have more important roles because it suppresses the oxidative burst and resistance of the host plant and triggers mediated apoptotic-like programmed cell death (PCD; Kim et al., 2008). In the compatible interactions between *S. sclerotiorum* and its host plant, host cells maintain viability and a suppression of the oxidative burst is observed, which is akin to compatible biotrophic pathogens during the early stage of infection (Williams et al., 2011; Kabbage et al., 2013, 2015). Furthermore, direct acidification within the middle lamella enhances the activity of many cell wall-degrading enzymes, including polygalacturonases (PGs; Riou et al., 1991). Secretion of oxalic acid may help inactivate plant PG-inhibiting proteins, thereby allowing the pathogen to overcome this specific host defense response (Favaron et al., 2004). Nonetheless, despite extensive studies on *S. sclerotiorum*, its pathogenic mechanisms are still incompletely understood because its pathogenesis is very complex (Hegedus and Rimmer, 2005; Amselem et al., 2011; Williams et al., 2011).

Several attempts to genetically study the pathosystem *S. sclerotiorum*–*P. vulgaris* have been performed to establish methods to detect physiological resistance (Kolkman and Kelly, 2000; Schwartz and Singh, 2013). The infection process and establishment of compatible interactions have also been well characterized at the cytological level (Lumsden, 1979; Lumsden and Wergin, 1980; Tariq and Jeffries, 1986). Differential accumulation of specific defense-related transcripts, such as mRNA for polygalacturonase-inhibiting protein (PGIP) and pathogen related proteins, during the *S. sclerotiorum*–*P. vulgaris* interaction has also been reported (Oliveira et al., 2010, 2013; Kalunke et al., 2011).

Increasing our knowledge of the infection strategies of necrotrophic pathogens, in general, and of *S. sclerotiorum*, specifically, should be helpful in designing novel rational disease control strategies. This study, in which genes differentially expressed in the *S. sclerotiorum*–*P. vulgaris* interaction were identified and analyzed, provides a starting point for achieving a better understanding of the pathosystem *S. sclerotiorum–*P. vulgaris.**

## Materials and Methods

### Plant Material and Pathogen Inoculation

Dry bean plants (*Phaseolus vulgaris* L. cv. BRS Pérola), which are susceptible to *S. sclerotiorum*, were grown in 2-kg plastic pots containing soil fertilized with NPK (4-39-16) (1 g/kg of soil) in a greenhouse at 24 ± 2°C with 60 ± 5% relative humidity and a 16/8 h light/dark period.

*Sclerotinia sclerotiorum* isolate SPS was collected from a naturally infected dry bean plant and grown on Petri dishes containing potato-dextrose agar (PDA) culture medium for 5 days at 20°C, and 3-mm plugs of this culture were used to inoculate the axillary region of dry bean plants at the flowering stage (R6), which is the main stage of infection in field. The control group was composed of a set of plants mock-inoculated with sterile agar plugs. All of the plants were kept at 20°C and 90% relative humidity to provide adequate conditions for infection. Tissue samples were collected from both the necrotic part and the chlorotic area of each lesion within 10 mm of the lesion edge at 6-, 12-, 24-, 48-, and 72-h post inoculation (hpi) and immediately frozen in liquid nitrogen prior to RNA and protein extraction. The experimental design was completely randomized and consisted of three biological replicates for each of the treatments. For each of the experimental conditions, stem segments (∼10 mm) from 20 plants different plants were pooled together to form one of three biological replicates.

### RNA Extraction

Total RNA was extracted from the plant tissues (stem segment) and fungal material using the standard Trizol protocol (Invitrogen Corp., Carlsbad, CA, USA). Total RNA samples were examined quantitatively using the Qubit fluorometry assay (Invitrogen Corp., Carlsbad, CA, USA) and qualitatively using agarose gel electrophoresis.

Two different RNA mixtures were prepared as the source of the tester and driver samples for the suppressive subtractive hybridization (SSH) library construction. For the tester samples, equal amounts of the total RNA isolated from the stem tissues inoculated with *S. sclerotiorum* at 6, 12, 24, 48, and 72 hpi were mixed. In the case of the driver samples, RNA from non-inoculated stem tissues harvested at the same times (6–72 h) plus RNA extracted from the mycelium of the fungus [grown separately in minimal medium (2 g/L NH_4_NO_3_, 1 g/L KH_2_PO_4_, 0.1 g/L MgSO_4_.7H_2_O, 0.5 g/L yeast extract, 3 g/L DL-malic acid, 1 g/L NAOH) supplemented with 1% glucose] were mixed at a 2:1 ratio (estimated based on qPCR experiments – data not shown).

### Suppressive Subtractive Hybridization Library Construction

For subtractive hybridization, 1.0 μg of each total RNA mixture was employed to produce double-stranded cDNA using the Super SMART^TM^ cDNA Synthesis Kit (Clontech Laboratories Inc., Palo Alto, CA, USA) according to the manufacturer’s instructions. To determine which genes were differentially expressed during the interaction with *S. sclerotiorum*, a forward subtractive cDNA library was constructed using the PCR-Select^TM^ cDNA Subtraction Kit (Clontech Laboratories Inc., Palo Alto, CA, USA) according to the standard protocol provided. The subtracted cDNA population was cloned into a PCR 4^®^-TOPO vector (Invitrogen Corp., Carlsbad, CA, USA) and used to transform One Shot^®^ Top 10 Eletrocomp^TM^ *Escherichia coli* cells (Invitrogen Corp., Carlsbad, CA, USA). The plasmid DNA was obtained using alkaline lysis. The cloned products were sequenced using a M13 forward primer and the Big Dye Terminator v 3.1 Cycle Sequencing Kit (Applied Biosystems, Carlsbad, CA, USA). The automated capillary electrophoresis sequencing runs were performed on an ABI prism 3130 analyzer (Applied Biosystems, Carlsbad, CA, USA).

### Bioinformatic Analyses of Expressed Sequence Tags (ESTs)

The sequences were pre-processed using Phred (Ewing and Green, 1998) and Cross Match software^1^. Sequences with at least 100 nucleotides and a Phred quality greater than or equal to 20 were considered for further analysis. Cleaned ESTs were assembled into contigs using the CAP3 assembly program and compared against the GenBank non-redundant (nr) database using the BLASTx algorithm available at the National Center for Biotechnology Information^2^. Functional annotation using Gene Ontology terms^3^ was analyzed with the Blast2GO program (Conesa and Götz, 2008). KEGG pathway annotation was performed using the Kyoto Encyclopedia of Genes and Genomes (KEGG) pathway database^4^.

Primers were designed using Software Primer Express (Applied Biosystems, Carlsbad, CA, USA) for the amplification of gene fragments that were approximately 80–150 bp in length and with an annealing temperature of 60°C (**Table 1**). The primer specificity was checked *in silico* against the NCBI database^2^ through the Primer-BLAST tool. The selected primers were tested with PCR, and their specificity was checked by nucleotide sequencing on an ABI 3130 sequencer (Applied Biosystems, Carlsbad, CA, USA) using DyeTerminator chemistry 4 to confirm their identities.

**Table 1:** Oligonucleotide primer pairs used for quantitative real-time polymerase chain reaction (RT-qPCR) analysis.

Gene	Description/Sequence name	Forward/Reverse
**Fungal Primers**		
*Sspg1*	Endopolygalacturonase 1	F: TCTTGCAGCAGTCGAGAAG R: GTGTTGTGTCCGAGGGAGT
*Sspg3*	Endopolygalacturonase 3	F: ACCCACCACTTTGGCTACTG R: TGAGACGGTAAGACCCTTG
*Sspg6*	Endopolygalacturonase 6	F: AAGCTTATTGGAATGGGTAT R: CTGGAGTTGACGATTTGACTA
*SsBgsidase*	Glucan endo-1,3-β-glucosidase/SS_038	F: CTGACGGTTCGACCCTGTAT R: GTACTCCCAACGGAGAACCA
*β-1,4*	β-1,4-glucanase/SS_041	F: CAAGGCAGCTTAACCGCTAC R: GATCCATCGGAGTCGAGGTA
*acp1*	Acid protease/SS_011	F: GCCACCCAAAACGGAGAAT R: GAGGTGAGGACGGAGTTTTGTT
*aspS*	Aspartyl protease/SS_014	F: TGCTACTGGGTCCAACATCGT R: TGCGCTTGATGCACTTGGT
*ScXylo*	β-xylosidase/SS_024	F: CGCTCTTTTTCCCCATACAA R: AGCGATGCGTATCTTCGAGT
*Cellobio*	Cellobiohydrolase/SS_091	F: ACTCTCTGCCCTGATGCAGT R: AGGAGTGTAAGCGGCAGAAA
*oah*	Oxaloacetate acetylhydrolase	F: CCCAATCGTCGAGGACAAGC R: TGCCTGCTCCGGTCATGTAA
*Perilipin*	Perilipin/SS_008	F: GAAATTCGGCAAGCCATTTA R: TGCCCTTTGAAATCGGATAG
*gpdh*	Glyceraldehyde-3-phosphate dehydrogenase	F: TGGCTCCTACTAAAGTTG R: CAAGCAGTTGGTTGTGCAAG
**Plant primers**		
*PvPR1*	Pathogenesis-related PR-1	F: AAAGCCAAGAGCGATTCTCTTTTCA R: GAACACTCTGATTTGATAACACTTC
*PvPR2*	B1-3 endoglucanase	F: GAAGATGAGCtCAAAGCTGGTAA R: CAAGGATTGGCCAAAAGGTA
*PvPR3*	Chitinase class I	F: ATTGTTGTGCCAATCCCTTT R: CACCGCCATACAGTTCAAAA
*PvPAL*	Phenylalanine ammonia-lyase	F: GACACACAAGTTGAAGCACCA R: TGCAGCTTCTTAGCATCCTTC
*PvLOX*	Lipoxygenase	F: AGCACTGTGCCTGTTTTCAGT R: AACACACGAGAAGATTCAACCA
*PvDOX*	α-dioxigenase	F: CACAAATCCTCCCAAAATGG R: AAAGGTTCACACCATTGATTGG
*PvFPS1*	Farnesyl pyrophosphate syntase 1	F: CGGAATAGACGTTGAGGAATG R: AACACCTACCCGAATTTCTGC
*PvISOF*	Isoflavonoid glucosyltransferase	F: AGCTGAAACACAGCACCAACT R: AACACCATGCTTGGCAAATAG
*Pvcallose*	Callose synthase-like protein	F: TGGCTTAGTATTCGGATGTACCA R: CGTTGTAATGAAAGCGAAGTGT
*Pv4CL*	4-coumarate CoA-ligase	F: AGGTTGTTGGTGCTGAGAATG R: CCAACCAAGTCAAAGATTCCA
*PvHPRP*	Hypersensitive-induced response protein	F: ATTGCATGGTTCATAGCCAGT R: CCTCCACACAAGTATCAAAGGA
*PvGST*	Glutatione *S*-transferase	F: AGCTCTTCAAGGACACTGAGCCAA R: AAAGGCTGTGGATGCTGCACTAGA
*PvPOD*	Peroxidase	F: TCCTTTTCAGCACTTTCACT R: AGAAAGCAGTGTTCTTGTGG
*Act11*	Actin11	F: TGCATACGTTGGTGATGAGG R: AGCCTTGGGGTTAAGAGGAG

### Reverse Transcriptase – quantitative PCR (RT-qPCR)

RNA was extracted from plant tissues and fungal material using the standard Trizol protocol (Invitrogen Corp., Carlsbad, CA, USA). After DNase I (Invitrogen Corp., Carlsbad, CA, USA) treatment in the presence of a RNase inhibitor (Invitrogen Corp., Carlsbad, CA, USA), equal amounts of RNA (1 μg) were reverse-transcribed using an oligo(dT)12–18 primer and evaluated with qPCR. RT-qPCR analysis was carried out using the StepOnePlus^TM^ System and Power SYBR^®^ Green PCR Master Mix (Applied Biosystems, Carlsbad, CA, USA) in 10 μL reactions containing 0.4 μM of each oligonucleotide (**Table 1**), 6 μL of SYBR Green PCR Master mix (2x), and 0.2 μL of template cDNA. After initial denaturation at 95°C for 10 min, amplifications were performed for 40 cycles at 95°C for 15 s and at 60°C for 1 min. To check the specificity of the PCR product, the melting curves were analyzed for each data point. The relative expression levels of the target genes were calculated using the ΔΔCt method (Livak and Schmittgen, 2001) and the reference genes *Actin-11* for *P. vulgaris* (Borges et al., 2012a) and *gpdh* for *S. sclerotiorum*. The reference sample was chosen to represent 1x expression of the gene of interest. Three samples were analyzed for each treatment. The values are expressed as the mean ± standard deviation (SD).

### Light Microscopy

Resin sections of stems inoculated with fungal samples (12–48 hpi) and without inoculation (control) were prepared for histological observation using light microscopy according to the method described by Mendoza et al. (1993). Briefly, the samples were dehydrated using a graded alcohol series, embedded in a mixture of absolute ethanol and resin (1:1, v/v; Historesin, Leica Microsystems Nußloch GmbH, Heidelberg, Germany) for 4 h, and stored for 24 h in pure Historesin. The resin was polymerized using a hardener, and the samples were sectioned into 8-μm thick slices with a rotary microtome. The sections were stained with 0.05% toluidine blue for 2 min and mounted. Tissue sections were photographed using a Leica DM500 microscope with a Leica ICC50 camera and the Leica Application Suite (LAS) EZ V3.0.0 software (Leica Microsystems, Switzerland).

## Results

### Disease Development: Time Course Analysis of *S. sclerotiorum* Infection

**FIGURE 1:**
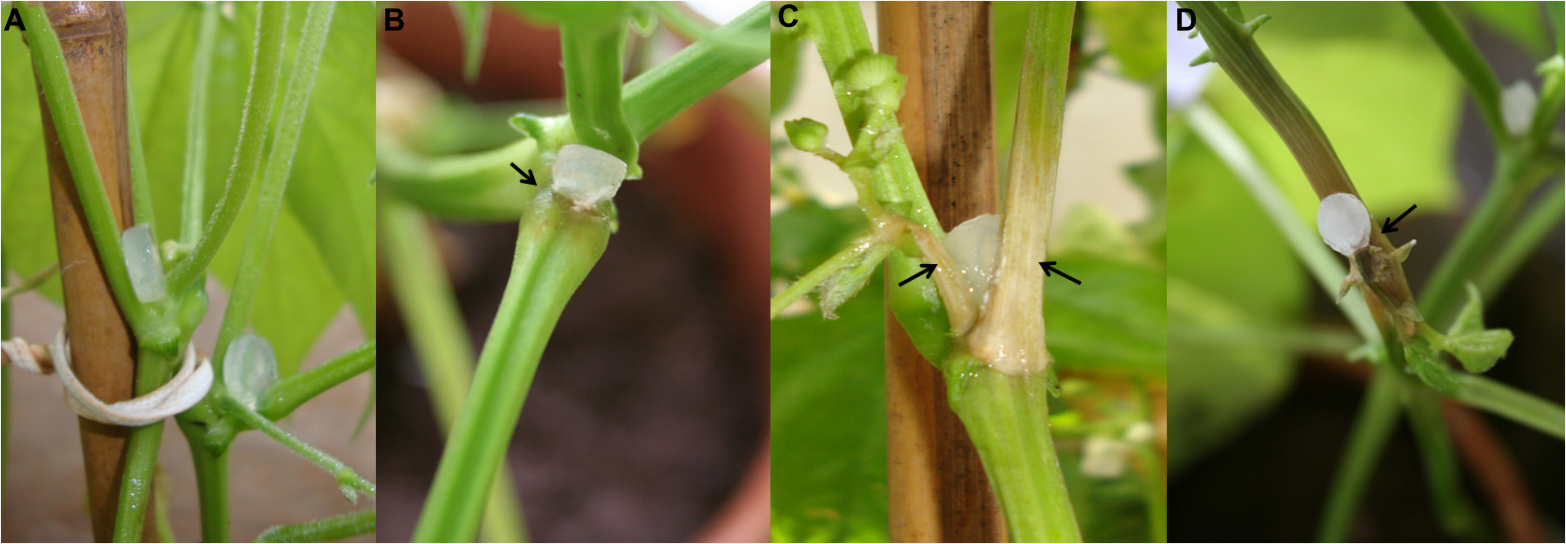
**Plant disease symptoms observed (arrow) in *Phaseolus vulgaris* stems inoculated with plugs of *Sclerotinia sclerotiorum* PDA culture. (A)** 12 hpi. **(B)** 24 hpi. **(C)** 48 hpi. **(D)** 72 hpi.

To evaluate the *S. sclerotiorum* behavior in the interaction with *P. vulgaris* cv BRS Pérola, stem tissues of inoculated plants were sampled at 6-, 12-, 24-, 48-, and 72-hpi. The pathogen showed an infection capacity at 12 hpi, although the tissue from the inoculated plants remained healthy (**Figure 1A**). Necrotic spots appeared on stem tissues after 24 hpi (**Figure 1B**), and small necrotic areas were clearly visible at 48 hpi (**Figure 1C**). In addition, at 72 hpi, late extensive necrosis was observed in the vascular tissues of inoculated stems (**Figure 1D**).

### Functional Classification of the Significantly Differentially Expressed Genes

Aiming to isolate the pathogen and plant genes that were differentially expressed during the *S. sclerotiorum*–*P. vulgaris* interaction, the susceptible common bean cultivar BRS Pérola was used, and suppression subtractive hybridization (SSH) was employed for the generation of a cDNA library. The SSH library was constructed using cDNA from stem tissues inoculated with *S. sclerotiorum* as the tester and cDNA from non-inoculated stem tissue plus RNA from the fungus grown in minimal medium as the driver.

A total of 1440 plasmids were sequenced. After removing the vector and adaptor sequences, the cleaned expressed sequence tags (ESTs) were assembled into contigs using the CAP3 assembly program comprising a total of 979 unigenes (representing 430 contigs and 549 singletons). The putative function of the 979 ESTs was assigned using the BLASTX program of the NCBI nr database employing the Blast2GO program with an *E-value* cutoff of <1^e-06^ and the Gene Ontology database.

**FIGURE 2:**
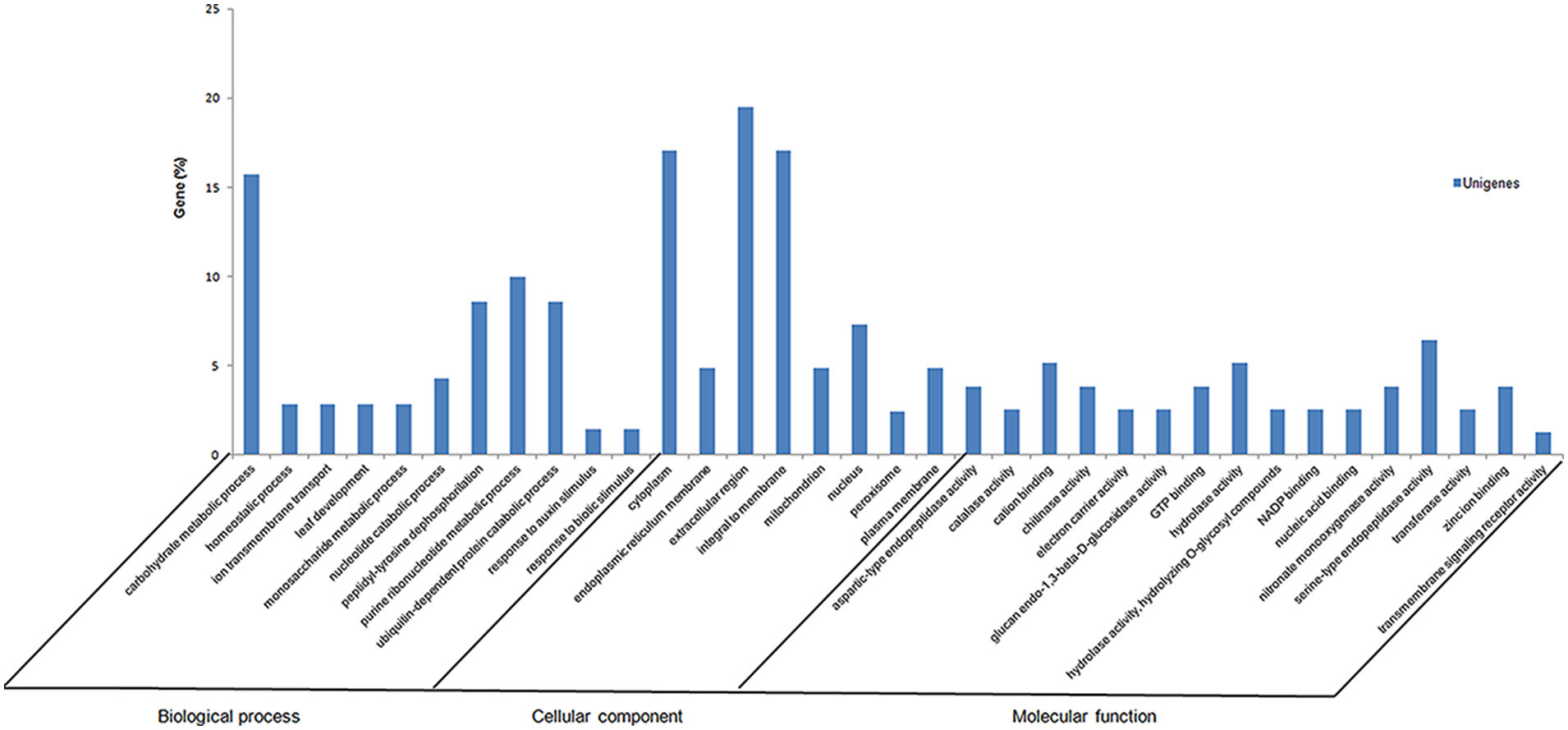
**Summary of Gene Ontology annotation as assigned by Blast2GO.** Functional annotation of the expressed sequence tags (ESTs) in *Phaseolus vulgaris* infected with *Sclerotinia sclerotiorum* SSH library using gene ontology terms (http://www.geneontology.org) by Blast2GO program. Most consensus sequences were grouped into major functional categories: biological process, cellular component, and molecular function.

Initially, to identify metabolic processes that are specifically modulated by fungal infection, the 979 unigenes were classified into functional categories (biological processes, cellular components, and molecular functions) according to their putative biological functions reported by the Gene Ontology database (**Figure 2**). Of 979 unigenes derived from the *S. sclerotiorum*–*P. vulgaris* interaction, 29% were categorized as fungal, and 58.3% as plant genes. Of these, 12.7% had no significant match and therefore could not be classified.

The SSH library was normalized to reduce the redundancy of the most highly expressed genes. Nevertheless, some genes are very highly expressed and thus remain overrepresented in the normalized library. The number of reads/contig and their length distribution are shown in Supplementary Table S1. Most contigs were assembled from two to three reads. As expected, the largest contigs (i.e., those composed of most sequences) are involved in stress response pathways, such as contig 161, which has 28 sequences encoding a binuclear zinc transcription factor (*Colletotrichum gloeosporioides*).

### Gene Expression Profiles in *S. sclerotiorum* during *P. vulgaris* Infection

A total of 979 unigenes (representing 430 contigs and 549 singletons) were generated from the *S. sclerotiorum*–*P. vulgaris* interaction and aligned to the *Sclerotinia* genome database (Broad Institute, Cambridge, MA, USA) for the identification and screening of fungal sequences. Preliminary analysis of the fungal unigenes (163 contigs and 120 singletons) revealed a total of 67 unique fungal ESTs in this database. The proteins encoded by this unique EST set were assigned to the following major categories: cell wall degradation, protein degradation, intracellular transport, oxidative stress, among others (**Table 2**). Global analysis detected a small but significant group of genes related to virulence during the penetration stages of development (6–72 hpi).

**Table 2:** Annotation of *Sclerotinia sclerotiorum* expressed sequence tags (ESTs) (contigs) derived from its interaction with *Phaseolus vulgaris*.

Contig	Accession number^a^	Name and/or putative function	Organism	*E* -value
**Cell wall degradation**				
Contig 27 – SS_024	SS1G_01493	β-xylosidase^b^	*S. sclerotiorum*	3e-15
Contig 45 – SS_041	SS1G_13255.3	β-1,4-glucanase^b^	*S. sclerotiorum*	6e-73
Contig 57 – SS_052	SS1G_08474	β-1,3-glucanase precursor	*S. sclerotiorum*	6e-05
Contig 98 – SS_091	SS1G_07146.3	Cellobiohydrolase^b^	*S. sclerotiorum*	2e-40
Contig 124 – SS_116	SS1G_12937	Glycosyl hydrolase	*S. sclerotiorum*	2e-32
Contig 145 – SS_136	SS1G_07393	β-1,3-glucanase	*S. sclerotiorum*	1e-64
Contig 42 – SS_038	SS1G_02789	Glucan endo-1,3-β-glucosidase^b^	*S. sclerotiorum*	3e-22
Contig 33 – SS_029	SS1G_01005	α-glucosidaseputative	*S. sclerotiorum*	3e-40
Contig 18 – SS_016	SS1G_12021	β-1,6-glucanase putative	*S. sclerotiorum*	5e-18
**Protein degradation**				
Contig 25 – SS_022	SS1G_0336	Serine peptidase putative	*S. sclerotiorum*	3e-43
Contig 26 – SS_023	SS1G_06534	Serin endopeptidase	*S. sclerotiorum*	2e-53
Contig 16 – SS_014	SS1G_03181	Aspartyl protease^b^	*S. sclerotiorum*	2e-20
Contig 13 – SS_011	SS1G_00624	Acid protease^b^	*S. sclerotiorum*	8e-15
Contig 62 – SS_057	SS1G_11818	Vacuolar protease A	*S. sclerotiorum*	4e-53
Contig 74 – SS_068	SS1G_00477	Ubiquitin homeostasis protein lub1 (phospholipase)	*S. sclerotiorum*	1e-12
Contig 63 – SS_058	SS1G_0770	Proteasome component PRE6	*S. sclerotiorum*	3e-47
Contig 61 – SS_056	SS1G_01331	Translocation protein Sec62 putative	*S. sclerotiorum*	1e-22
**Intracellular transport**				
Contig 40 – SS_036	SS1G_07860	Protein SEY1	*S. sclerotiorum*	6e-42
Contig 77 – SS_071	SS1G_09620	Hypothetical protein (GTPase)	*S. sclerotiorum*	2e-04
Contig 103 – SS_096	SS1G_10229	GTP-binding protein EsdC	*S. sclerotiorum*	1e-51
Contig 160 – SS_150	SS1G_08784	GTP-binding protein	*S. sclerotiorum*	6e-33
Contig 148 – SS_139	SS1G_11149	SEC24 related gene family (GTPase)	*S. sclerotiorum*	3e-31
Contig 9 – SS_007	SS1G_02490	DENN domain-containing protein	*S. sclerotiorum*	2e-32
Contig 86 – SS_080	SS1G_09635	Geranylgeranyl pyrophosphate synthetase (biosynthesis of secondary metabolites)	*S. sclerotiorum*	5e-25
**Oxidative stress**				
Contig 53 – SS_048	SS1G_14466	Oxidoreductase, 2-nitropropane dioxygenase family	*S. sclerotiorum*	9e-19
Contig 44 – SS_040	SS1G_12928	Peroxidase/catalase	*S. sclerotiorum*	2e-38
Contig 88 – SS_082	SS1G_10037	Cytochrome P450	*S. sclerotiorum*	4e-29
Contig 161 – SS_151	SS1G_06754	Zinc transcription factor	*S. sclerotiorum*	4e-42
Contig 153 – SS_143	SS1G_11235	2-nitropropane dioxygenase	*S. sclerotiorum*	2e-44
**Others**				
Contig 10 – SS_008	SS1G_06319	Perilipin MPL1- like protein CAP 20^b^	*S. sclerotiorum*	1e-07
Contig 97 – SS_090	SS1G_03197	pH-response regulator protein	*S. sclerotiorum*	2e-09
Contig 75 – SS_069	SS1G_10538	Ceramidase	*S. sclerotiorum*	6e-20
Contig 142 – SS_133	SS1G_00477	Phospholipase	*S. sclerotiorum*	1e-12

*^a^ GenBank database (http://www.ncbi.nlm.nih.gov)*

*^b^ Validated by RT-qPCR*

To gain a better understanding of the mechanisms of *S. sclerotiorum* pathogenesis, 9 genes from the *S. sclerotiorum–P. vulgaris* SSH library related to plant cell wall degradation were selected for expression patterns analysis: PGs (*Sspg1, Sspg3*, and *Sspg6*); cellulases (β-1,3-glucosidase, β-1,4-glucanase, and cellobiohydrolase); hemicellulases (β-xylosidase); and proteases (aspartyl protease – *aspS* and acid protease – *acp1*). These genes were evaluated at 6, 12, 24, 48, and 72 hpi by reverse transcription quantitative real-time polymerase chain reaction (RT-qPCR). The relative expression levels of all genes tested were normalized to the *S. sclerotiorum gpdh* gene.

**FIGURE 3:**
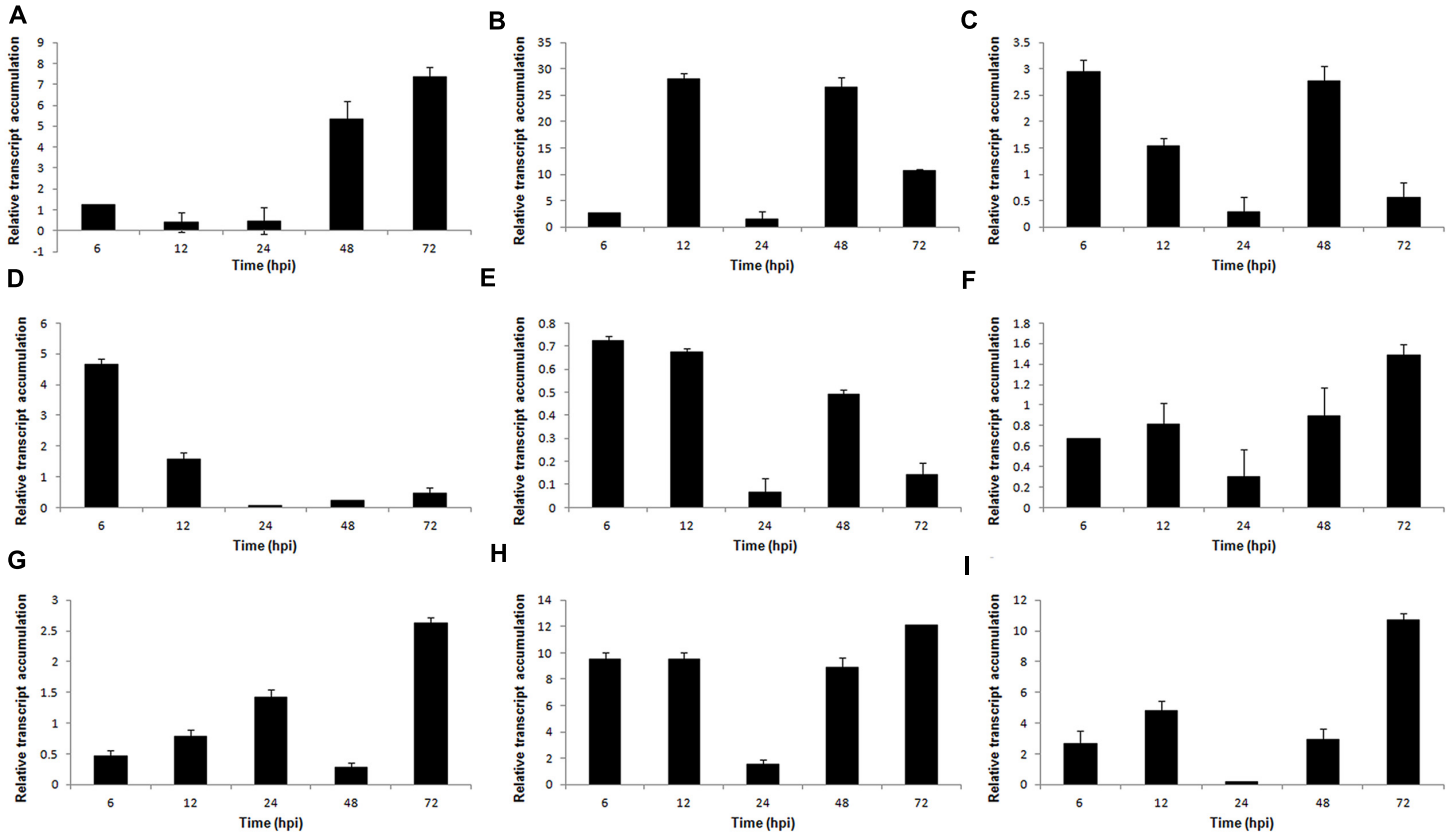
**Gene expression profiles of *S. sclerotiorum* during infection to *P. vulgaris.* Expression analyses using RT-qPCR were performed and transcript levels were calculated in triplicate using a comparative method.** *GPDH* gene was used as the reference gene in *S. sclerotiorum* and a health plant as the reference sample. Tissue samples were collected at 6, 12, 24, 48, and 72 hpi. Results are reported as means ± standard deviation (SD) of three samples for each treatment. **(A)** *Sspg1*. **(B)** *Sspg3*. **(C)** *Sspg6*. **(D)** *β-1,3-glucosidase*. **(E)** *β-xylosidase.* **(F)** *β-1,4-glucanase*. **(G)** cellobiohydrolase. **(H)** *acp1* (acid protease). **(I)** *aspS* (aspartyl protease).

The PG *Sspg1* gene showed higher transcript levels in the late stage of the interaction. *Sspg3* expression was induced at 12 hpi and was reduced at 72 hpi. The *Ssp6* gene showed higher transcript levels at the early stages (6–12 hpi) after inoculation (**Figures 3A–C**). Previously, we showed that the *Sspg1* and *Sspg5* genes are highly expressed during *S. sclerotiorum* infection of *P. vulgaris* plants and also that the *Sspg3* gene is expressed during the initial phase of colonization, whereas *Sspg6* is weakly expressed during this process (Oliveira et al., 2010).

The genes encoding β-1,3-glucosidase and β-xylosidase were also upregulated during the early stages of the *S. sclerotiorum–P. vulgaris* interaction (**Figures 3D,E**). Conversely, the genes encoding β-1,4-glucanase and cellobiohydrolase showed increased expression levels in the later stages of infection (**Figures 3F,G**). The gene *acp1*, which encodes an acid protease, was highly expressed from the beginning of infection, and presented the highest level of expression at 72 hpi. A similar profile was also observed for the *aspS* gene, which encodes an aspartyl protease (**Figures 3H,I**).

**FIGURE 4:**
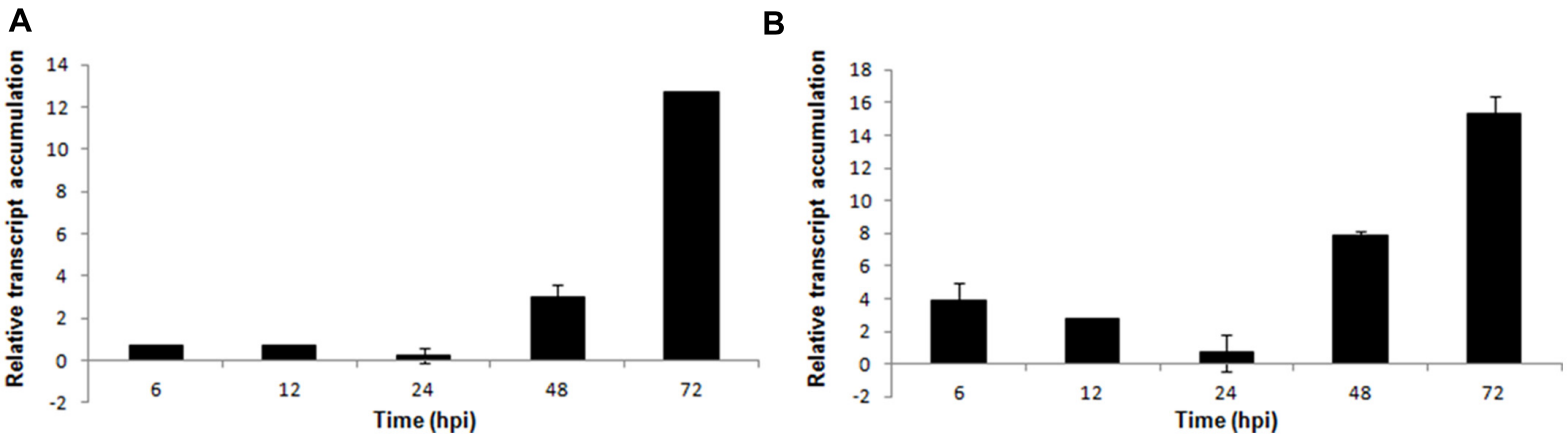
**Gene expression profiles of *S. sclerotiorum* during infection to *P. vulgaris*.** Expression analyses using RT-qPCR were performed and transcript levels were calculated in triplicate using a comparative method. *GPDH* gene was used as the reference gene in *S. sclerotiorum* and a health plant as the reference sample. Tissue samples were collected at 6, 12, 24, 48, and 72 hpi. Results are reported as means ± standard deviation (SD) of three samples for each treatment. **(A)** *oah* (oxaloacetate acetylhydrolase). **(B)** *perilipin*.

In *S. sclerotiorum*, as well as in other fungi, oxalic acid is produced from oxaloacetate in a reaction catalyzed by oxaloacetate acetylhydrolase (OAH). In this work, *oah* transcripts were detected in the early stage of infection (6 hpi), and its expression was higher in the late stage (72 hpi; **Figure 4A**).

Additionally, 15 unigenes (5 contigs and 10 singlets) that encode perilipin-like protein were detected in the *S. sclerotiorum–P. vulgaris* SSH library. Perilipin-like protein is largely responsible for the regulation of lipid storage and metabolism in filamentous fungi (Duan et al., 2013). Our analysis showed that although this gene was upregulated starting at the early stage of infection, the highest expression level was observed in the final stage (72 hpi; **Figure 4B**).

### Defense Genes Expressed and Regulated Over Time in the Common Bean Stem During Interaction with *S. sclerotiorum*

As a result, the majority of the cDNAs from the SSH library were derived from *P. vulgaris*. In total 570 unigenes (267 contigs and 303 singletons) showed significant similarities to plant sequences, that indicates that 58.3% (979 unigenes) of our database represent common bean genes. The list of *P. vulgaris* the most common contigs/reads and their length distribution are shown in Supplementary Table S1. The proteins encoded by the unique EST set were assigned to five major categories: metabolism, energy, protein degradation, response to biotic and abiotic stimulus, and others. An important group of the identified genes are related to biotic and abiotic stress responses, among these are PR1 (pathogenesis related protein 1), and peroxidase genes. Although most of the defense-related genes were not detected in our EST collection (979 unigenes), primers were designed based on the bean transcriptome sequence (Melotto et al., 2005) to investigate the plant response during the interaction with *S. sclerotiorum.* We searched for ESTs that may play an important role in the defense response to *S. sclerotiorum* disease and can be used as reference for comparison among different pathosystems.

We evaluated the temporal expression profile of the three main categories of defense-related genes, namely: (1) pathogenesis-related (PR) genes *PvPR1* (function unknown), *PvPR2* (β-1,3-endoglucanase), and *PvPR3* (chitinase); (2) phenylpropanoid pathway genes, such as *PvISOF* (isoflavonoid glucosyltransferase), *PvFPS1* (farnesyl pyrophosphate synthetase 1), *PvPAL* (phenylalanine ammonia-lyase), and *Pv4CL* (4-coumarate CoA-ligase), which are involved in phytoalexin biosynthesis; and (3) genes involved in defense and stress-related categories, such as *PvLOX* (lipoxygenase), *PvHIPRP* (hypersensitive-induced response protein), *PvGST* (glutathione *S*-transferase), *PvPOD* (peroxidase), and *PvDOX* (alpha-dioxygenase). The time-course expression patterns were determined using control and infected samples at 6, 12, 24, 48, and 72 hpi. The relative expression levels of all of the genes were normalized using the *P. vulgaris Actin 11* gene (*Act11*) as the reference gene (Borges et al., 2012a).

**FIGURE 5:**
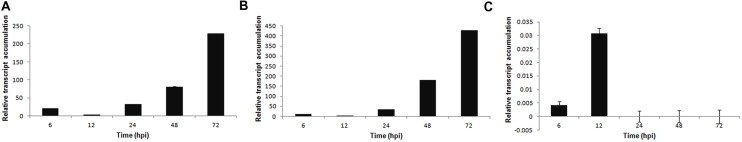
**Expression pattern of pathogenesis-related genes in *P. vulgaris* in response to *S. sclerotiorum* infection.** Expression analyses using RT-qPCR were performed and transcript levels were calculated in triplicate using a comparative method. *Actin-11* gene was used as the reference gene in *P. vulgaris* and a health plant as the reference sample. Tissue samples were collected at 6, 12, 24, 48, and 72 hpi. Results are reported as means ± standard deviation of three samples for each treatment. **(A)** Pv*PR1* (pathogenesis related protein 1). **(B)** *PvPR2* (pathogenesis related protein 2). **(C)** *PvPR3* (pathogenesis related protein 3).

Significant fold-change differences in expression levels were observed for all of the selected genes in the infected plant (fungus-inoculated) compared with the mock-inoculated plant. The temporal expression of the PR genes *PvPR1* (function unknown) and *PvPR2* (β-1,3-endoglucanase) showed a similar expression profile. Both genes presented a basal expression during the early stages of infection (6–12 hpi) and a high expression level at 72 hpi; however, at this time, the expression of *PvPR2* was two-fold higher than that of *PvPR1* (**Figures 5A,B**). The expression of the *PvPR3* gene was observed only in the early stages of infection (6–12 hpi), but at lower levels compared with the other *PvPR* genes analyzed (**Figure 5C**).

**FIGURE 6:**
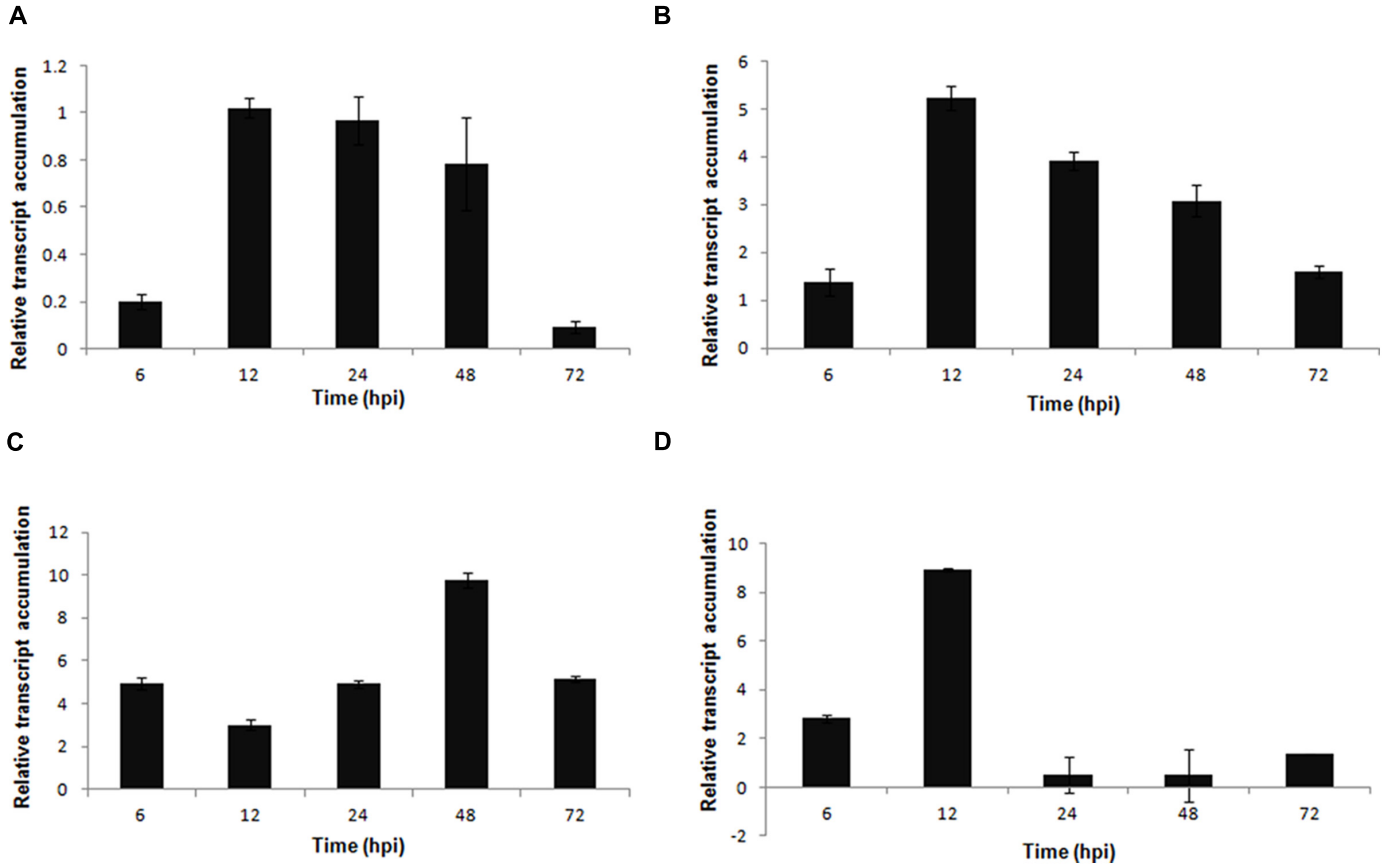
**Expression pattern of phenylpropanoid pathway genes in *P. vulgaris* in response to *S. sclerotiorum* infection.** Expression analyses using RT-qPCR were performed and transcript levels were calculated in triplicate using a comparative method. *Actin-11* gene was used as the reference gene in *P. vulgaris* and a health plant as the reference sample. Tissue samples were collected at 6, 12, 24, 48, and 72 hpi. Results are reported as means ± standard deviation of three samples for each treatment. **(A)** *PvISOF* (isoflavonoid glucosyltransferase). **(B)** *PvFPS1* (farnesyl pyrophosphate synthetase 1). **(C)** *PvPAL* (phenylalanine ammonia-lyase). **(D)** *Pv4CL* (4-coumarate CoA-ligase).

The genes involved in the phenylpropanoid pathway, *PvISOF* (isoflavonoid glucosyltransferase), *PvFPS1* (farnesyl pyrophosphate synthetase 1), *PvPAL* (phenylalanine ammonia-lyase), and *Pv4CL* (4-coumarate CoA-ligase), were upregulated in dry bean plants during the interaction with *S. sclerotiorum*. The highest levels of expression were registered for *PvPAL* (a two-fold increase compared with *PvFPS1* and a 10-fold increase compared with *PvISOF* at 48 hpi (**Figures 6A–C**) and *Pv4CL* at 12 hpi (**Figure 6D**).

**FIGURE 7:**
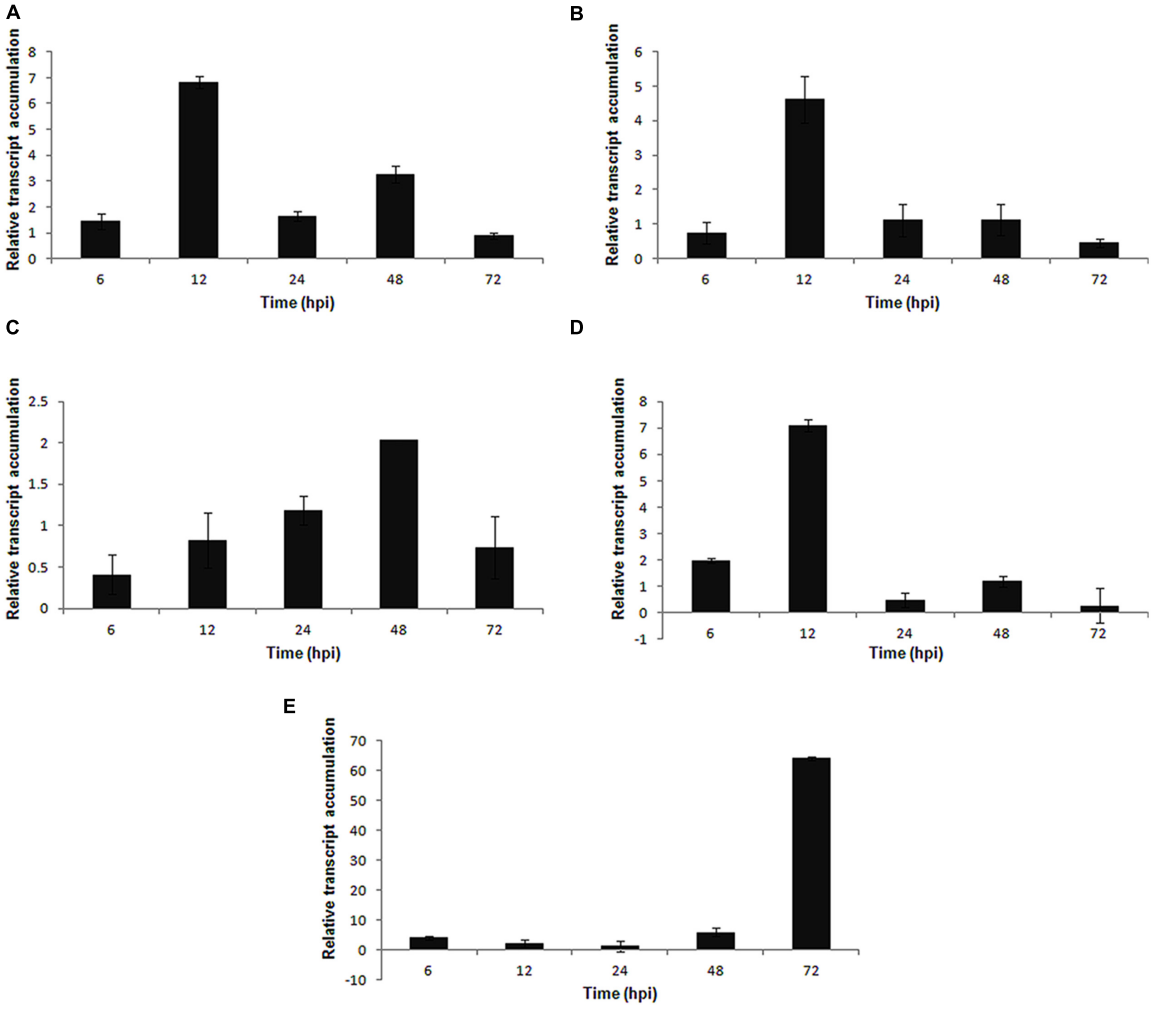
**Expression pattern of defense and stress related genes in *P. vulgaris* plant in response to *S. sclerotiorum* infection.** Expression analyses by RT-qPCR were performed and transcript levels were calculated in triplicate using a comparative method. *Actin-11* gene was used as the reference gene in *P. vulgaris* and a health plant as the reference sample. Tissue samples were collected at 6, 12, 24, 48, and 72 hpi. Results are reported as means ± standard deviation of three samples for each treatment. **(A)** *PvLOX* (lipoxygenase). **(B)** *PvHIPRP* (hypersensitive-induced response protein). **(C)** *PvGST* (glutathione *S*-transferase). **(D)** *PvPOD* (peroxidase). **(E)** *PvDOX* (alpha-dioxygenase).

Among the genes involved in the defense and stress-related categories, *PvLOX* was activated in the early stages of infection and reached a maximum expression at 12 hpi (**Figure 7A**). The *PvHIPRP* gene, which is related to hypersensitive responses (HR), was induced at the early stages of infection and peaked in expression at 12 hpi (**Figure 7B**). The *PvGST* (glutathione *S*-transferase) gene presented a progressive increase in expression up to 48 hpi, followed by a decrease at 72 hpi (**Figure 7C**), whereas the *PvPOD* (peroxidase) gene had a maximum expression at 12 hpi (**Figure 7D**). Finally, the *PvDOX* gene, which encodes α-dioxygenase, showed a high level of expression at 72 hpi (**Figure 7E**) in the *S. sclerotiorum*–*P. vulgaris* pathosystem.

**FIGURE 8:**
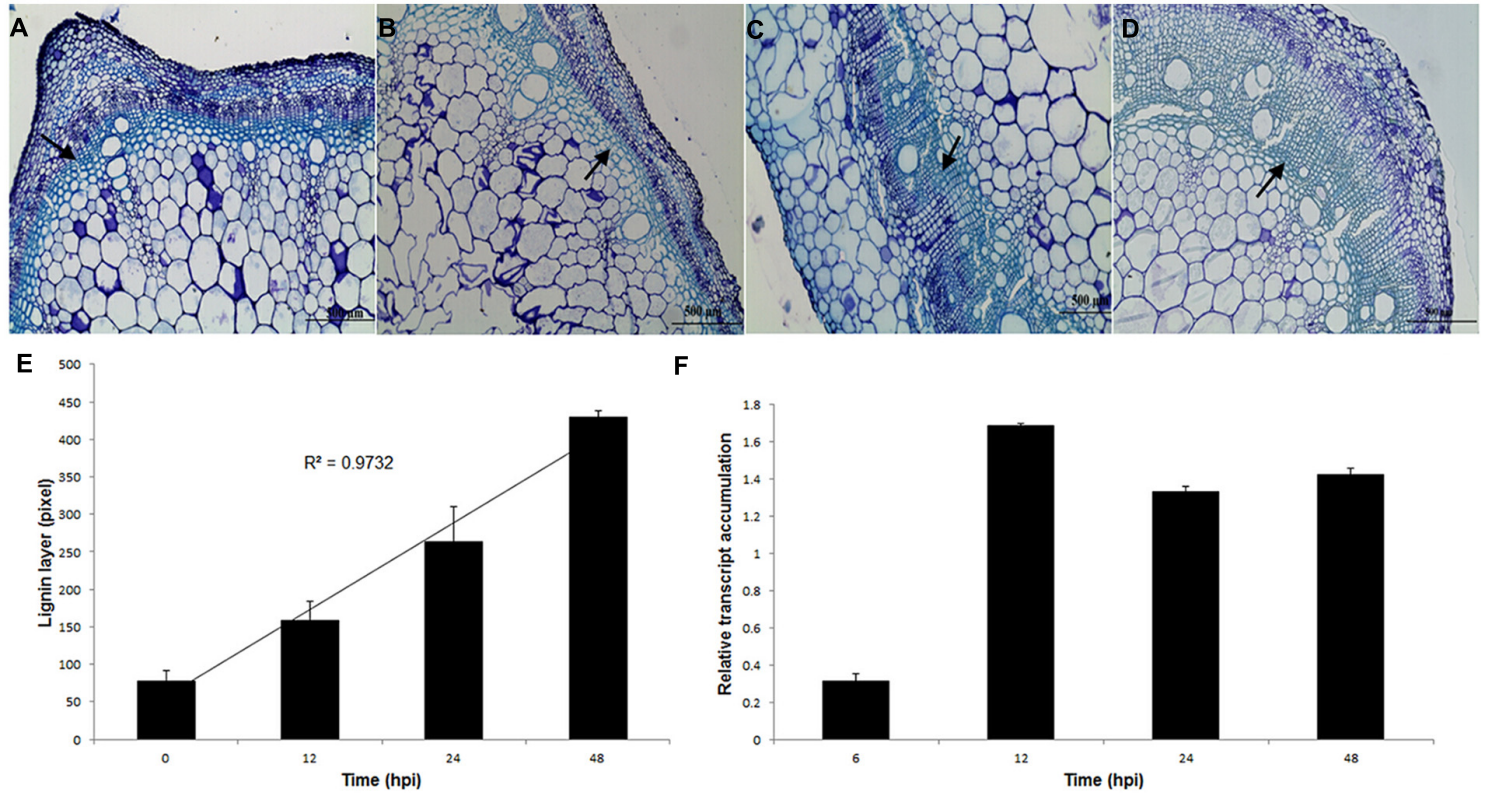
**Thickening layers of lignin in *P. vulgaris* stems in response to *S. sclerotiorum* infection. (A)** Without infection. **(B)** 12 hpi. **(C)** 24 hpi. **(D)** 48 hpi. Bar = 500 μm. **(E)** Thickness of lignin layer (arrows) measured using Leica LAS EZ V3.0.0 software (Leica Application Suite, Leica Microsystems, Switzerland). **(F)** Relative transcript accumulation of *Pvcallose* gene. Expression analyses using RT-qPCR were performed and transcript levels were calculated in triplicate using a comparative method. *Actin-11* gene was used as the reference gene in *P. vulgaris* and a health plant as the reference sample. Results are reported as means ± standard deviation of three experiments.

Distinct vascular changes in the stem were noticed in response to *S. sclerotiorum* infection. Histological sections of bean stem showed that under *S. sclerotiorum* infection (12–72 hpi; **Figures 8B–D**), bean plants had evident lignification, which was not observed in the (**Figure 8A**) control (mock-inoculated). The uppermost 3–4 cell layers of the epidermis were stained a greenish–blue color with Aniline blue, indicating an accumulation of phenolic compounds. Thickness of lignin layer (**Figure 8E**) was measured using Leica LAS EZ V3.0.0 software (Leica Application Suite, Leica Microsystems, Switzerland).

To assess whether the callose gene (*Pvcallose*) was modulated during the *S. sclerotiorum* invasion of *P. vulgaris* stem tissues, the level of its transcription was evaluated using RT-qPCR. The results indicate that this gene was activated in the early stages of infection (6 hpi) and showed an increased accumulation of transcripts from 12 hpi until 48 hpi (**Figure 8F**).

## Discussion

**FIGURE 9:**
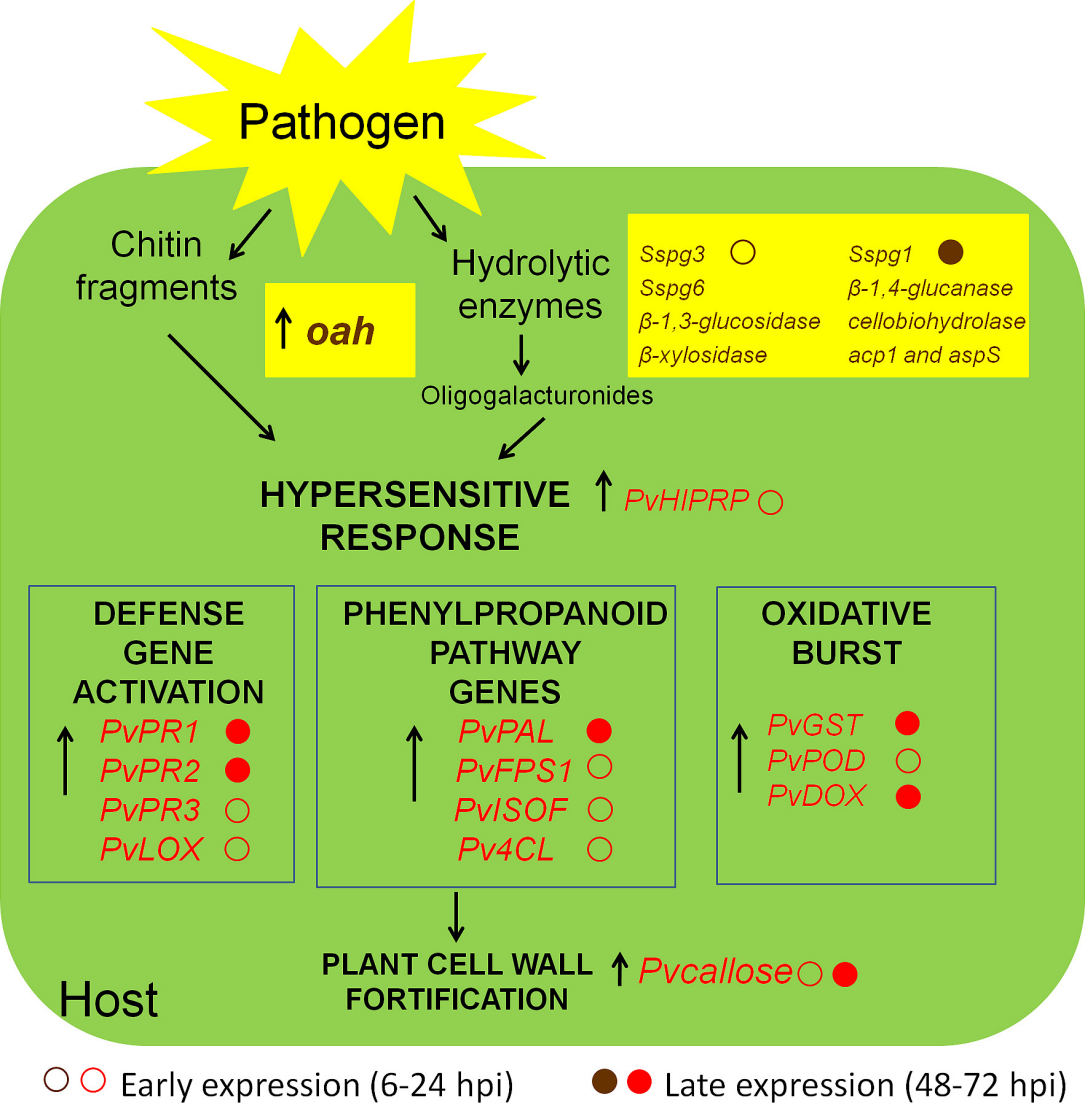
**Schematic representation of the interaction between *S. sclerotiorum* and *P. vulgaris.*** In the beginning of the pathogenic process, the fungus secretes hydrolytic enzymes (polygalacturonases, PGs) and oxalic acid. PGs degrade plant cell walls and release oligogalacturonides (OGs), which are elicitors of defense response. The hypersensitive response (HR) of the plant is used by the fungus as a strategy to promote virulence. Chitin fragments of fungus and OGs released from the plant itself allow the recognition of the presence of the pathogen and activate transcription factors which lead to the expression of genes belonging to different pathways of defense. The arrows indicate genes up-regulated in RTqPCR analysis. In brown, *S. sclerotiorum* genes, and in red, plant response genes.

Although *S. sclerotiorum* is considered to be a typical necrotroph, there is evidence that it colonizes plant tissues through multiple phases involving important transcriptional and physiological reprogramming (Hegedus and Rimmer, 2005; Kabbage et al., 2013, 2015; Guyon et al., 2014). In this study, we evaluated the infective process of *S. sclerotiorum* in *P. vulgaris* plants. The gene expression profiles of stem tissues in the inoculation site were examined over 3 days. The steady-state levels of all of the identified genes were quantified at the time of inoculation (0 hpi), at 2 early time points following inoculation (12 and 24 hpi) and at somewhat later time points (48 and 72 hpi), at which point necrotic lesions develop on the stems and intensive mycelial colonization occurs. From our transcript profile results, we propose the model illustrated in **Figure 9**, which depicts a plausible interpretation of the observed expression patterns of the *S. sclerotiorum*–*P. vulgaris* interaction.

The recent description of the genome sequences of two fungal necrotrophs, *S. sclerotiorum* and *Botrytis cinerea*, revealed 346 and 367 genes, respectively, encoding putative carbohydrate-active enzymes (CAZymes), with over 100 genes potentially associated with plant cell wall degradation (Amselem et al., 2011). In this work, we observed that many genes encoding cell wall-degrading enzymes: PGs (*Sspg1, Sspg3*, and *Sspg6*), cellulases (β-1,3-glucosidase, β-1,4-glucanase, and cellobiohydrolase) and hemicellulases (β-xylosidase), were found to be differentially expressed and showed accumulated transcripts in the inoculated sample.

A >2-fold induction in expression was measured at 12 hpi for *Sspg3* (28-fold), *Sspg6*-encoding endo-PGs (3-fold) and β-1,3-glucosidase genes (4.8-fold), even though no necrotic symptoms in the stem are visible at this time. PGs enable a pathogen to invade plant tissues; however, on the other hand, their activity may also trigger plant defense responses. Namely, the degradation of homogalacturonan, the main component of pectin, by PGs results in the release of oligogalacturonides (OGs), which are capable of inducing plant immune reactions. The inactivation of PG activity by plant PGIPs has been shown to reduce disease development (Powell et al., 2000). More recent attempts to delay disease by expressing PGIPs, which are plant proteins that inhibit fungal endopolygalacturonases, have been successful against neurotropic fungi (Borras-Hidalgo et al., 2012).

The increase in *β-1,4-glucanase* (1.5-fold) and *cellobiohydrolase* (2.8-fold) transcript accumulation coincided with the phase of symptom development (48 – 72 hpi) in which intensive mycelial colonization of the stem occurred and soaked lesions were observed.

Proteases secreted by this fungus can degrade host plant proteins, some of which are involved in defense responses to fungal inoculation, and also seem to be correlated with symptom development (Bolton et al., 2006). During the *S. sclerotiorum–P. vulgaris* interaction the expression of the gene (*acp1*), encoding an acid protease, and the gene *aspS*, encoding an aspartyl protease, was low at the beginning of infection but increased at the stage of spreading necrosis. Proteases are also regarded as antagonists of the antifungal proteins secreted as part of the defense response by the host. In previous reports, Poussereau et al. (2001) showed that *acp1* and *aspS* were expressed *in planta* during sunflower cotyledon infection.

In this work, oxaloacetate acetylhydrolase (*oah*) transcripts were already detected in the early stages of infection (6 hpi), and its expression was increased at the late stage of infection (72 hpi) (**Figure 4A**). Corroborating this, Liang et al. (2014) recently demonstrated that the Ss-*oah1* gene, which encodes an oxaloacetate acetylhydrolase, is required for oxalic acid accumulation and affects the pH-responsive growth, morphogenesis and virulence of *S. sclerotiorum* on a variety of plant hosts. Recently, oxalic acid was found to create reducing conditions in plant cells ahead of advancing hyphae. It was speculated that reductive conditions dampen the oxidative burst, allowing for precious time for fungal establishment prior to plant recognition (Kim et al., 2008). Also Kabbage et al. (2013, 2015) suggested that *S. sclerotiorum*, a prototypical necrotroph, has a biotrophic phase that occurs during the initial stages of disease establishment. During the early stages, oxalate dampens the host oxidative burst, an early response associated with plant defense, as part of a potential biotrophic interaction, before triggering generation of reactive oxygen species (ROS) at the later stages of infection, culminating in PCD, the advanced necrotrophic phase of the interaction and disease.

The results presented in this study show that dry bean plants respond to inoculation with *S*. *sclerotiorum* in the early stages of the process. The *PvHIPRP* gene, related to HR, was induced at the early stages of infection in infected tissue and had its expression peak at 12 hpi (4.8-fold). The importance of the HR for successful infection by necrotrophic pathogens is exemplified by studies showing that plants unable to undergo PCD (Dickman et al., 2001) or incapable of generating a HR (Govrin and Levine, 2000) are more resistant to such pathogens.

Among the genes involved in the defense and stress-related categories, *LOX* was activated in the early stages of infection (12 hpi – 6.8-fold). Lipoxygenase (LOX) catalyzes the oxidation of unsaturated fatty acids, producing oxylipins, which play a pivotal role in plant defense by acting as signaling molecules and/or protective compounds (Blée, 2002).

Plants that have to cope with oxidative stress can improve their ROS scavenging capacity via the upregulation of related enzymatic activities, such as glutathione *S*-transferase (Mhamdi et al., 2010), catalase, superoxide dismutase, and peroxidase (Nanda et al., 2010). In the present study, *PvPOD* (peroxidase) was induced at 6 hpi and peaked in expression at 12 hpi (7.5-fold). The *PvGST* (glutathione *S*-transferase) and *PvDOX* (α-dioxygenase) genes presented a progressive increase in expression following infectious development. The plants lacking a dioxigenase, DOX2 (α-DOX2-deficient mutant), which catalyzes oxylipin production from fatty acids, were more susceptible to *Botrytis cinerea* (Angulo et al., 2015). During the interaction with *Rhizoctonia solani*–*P. vulgaris*, this gene was highly expressed, and it was suggested that this enzyme protects plant tissues undergoing excessive necrosis associated with oxidative stress during pathogenesis (Guerrero-González et al., 2011).

Once the plant has identified the attack, it can respond by activating the synthesis of a diverse number of proteins. These proteins include antibiotic proteins, such as PR proteins. The genes encoding the *PvPR1* and *PvPR2* protein families increased in expression 250-fold and 450-fold at 72 hpi, respectively, following fungal infection. Similarly, Borges et al. (2012b) reported the upregulation of *PvPR1* and a β-1,3-glucanase (*PvPR2*) during the incompatible interaction between the common bean and *Colletotrichum lindemuthianum.* Although this interaction is not the same, we found a similar expression profile in our experiment in which *PvPR1* and *PvPR2* were activated in the later stages of infection.

PR1 proteins have frequently been used as markers for systemic-acquired resistance (SAR) and have been associated with antifungal properties, such as the hydrolysis of fungal cell walls. These signaling events occur normally by hormones such as salicylic acid (SA), ethylene and jasmonic acid (JA), which can activate PR genes (Edreva, 2005; Ferreira et al., 2007; Leon-Reyes et al., 2009).

The expression of the *PvPR3* gene (chitinases) was observed only in the early stages of infection (6–12 hpi). *PvPR3*, like *PvPR2* (β-1,3-endoglucanase), may act directly by inhibiting the growth of the pathogen or indirectly by aiding in the generation of signaling molecules that may function as elicitors of further defensive mechanisms (Edreva, 2005; Van Loon et al., 2006; Ferreira et al., 2007).

Apart from inducible proteins, active plant defenses against pathogens also include phenylpropanoid pathway enzymes, which are known to be involved in plant disease resistance. The comparative analysis of the bean plant inoculated with *S. sclerotiorum* and the mock-inoculated plant (without infection) proved that the *PvISOF* (isoflavonoid glucosyltransferase), *PvFPS1* (farnesyl pyrophosphate synthetase 1), *PvPAL* (phenylalanine ammonia-lyase), and *Pv4CL* (4-coumarate CoA-ligase) genes, which are involved in the phenylpropanoid pathway, were upregulated during infectious development.

PAL is the primary enzyme involved in the phenylpropanoid biosynthetic pathway and is responsible for the production of phenolic compounds, such as flavonoids, phytoalexins, lignins, and benzoic acid derivatives (Zhao et al., 2007). Several studies have demonstrated that the levels of PAL activity have important functions in the plant defense response to pathogen infection. *PvPAL* gene expression was induced by anthracnose fungal attack in *P. vulgaris* (Borges et al., 2012b), and PAL-suppressed plants were more susceptible to fungal infection (Maher et al., 1994).

In this study, the expression level of the *Pv4CL* gene was transient during the analyzed time period, with the greatest upregulation (9.5-fold) in the early stages (12 hpi) of infection. The 4-coumarate CoA-ligase is the third enzyme of the phenylpropanoid pathway in plants, and leads to the synthesis of lignin, pigments, and many defense molecules (Zabala et al., 2006).

In accordance with these findings, the results obtained here with aniline blue staining showed a progressive thickening of the lignin layers in dry bean plants during *S. sclerotiorum* infection. Certain phenylpropanoid compounds are polymerized to form defensive barriers, such as lignin, which is a crucial component of the plant defense repertoire against abiotic and biotic stress factors (Dixon et al., 2002). It has been strongly demonstrated that lignification is a common phenomenon in the expression of disease resistance in plants. Lignin synthesis is induced in response to mechanical damage or wounding, and many plants respond to invading pathogens with the deposition of lignin and lignin-like material (Vance et al., 1980; Nicholson and Hammerschmidt, 1992; Boudet et al., 1995; Dixon and Paiva, 1995). Additionally, callose is an effective barrier induced at the site of attack during the early stages of pathogen invasion and is an established marker associated with incompatible responses (Williams et al., 2011).

## Conclusion

This study contributes to a better understanding of the kinetics of induced defenses against a fungal pathogen of the common bean, as well fungal genes related to pathogenicity and virulence, and provides a valuable first step toward the understanding and analysis of the *Phaseolus vulgaris* – *S. sclerotiorum* interaction.

## Author Contributions

MO carried out the experiments and drafted the manuscript. RA participated in the transcription analysis experiments. MG participated in the design of the study and drafted the manuscript. SP conceived the study and participated in its design and coordination. All authors read and approved the final manuscript.

## Conflict of Interest Statement

The authors declare that the research was conducted in the absence of any commercial or financial relationships that could be construed as a potential conflict of interest.

## Footnotes

1 www.phrap.org

2 http://www.ncbi.nlm.nih.gov

3 http://www.geneontology.org

4 http://www.genome.jp/kegg
