# Supplementary material for: Analysis of genes that are differentially expressed during the Sclerotinia sclerotiorum–Phaseolus vulgaris interaction
Source: Front Microbiol. 2015 Oct 26;6:1162. doi: 10.3389/fmicb.2015.01162 (PMC4620421; doi:10.3389/fmicb.2015.01162)
Supplement: Supplementary file 1 [file Table_1.DOCX]

***Supplementary Material***

**Analysis of genes differentially expressed during *Sclerotinia sclerotiorum*–*Phaseolus vulgaris* interaction**

Marília Barros Oliveira^1^, Rosângela Vieira de Andrade^2^, Maria Fátima Grossi-de-Sá^2,3^ and Silvana Petrofeza^1*^

^1^Instituto de Ciências Biológicas, Universidade Federal de Goiás, Goiânia, Brazil

^2^Programa de Pós-graduação em Ciências Genômicas e Biotecnologia, Universidade Católica de Brasília, Brasília, Brazil

^3^Embrapa Recursos Genéticos e Biotecnologia, Laboratório de Interação Molecular Planta-Praga, Brasília, Brazil

*Correspondence: Silvana Petrofeza, Departamento de Bioquímica e Biologia Molecular, Instituto de Ciências Biológicas, Caixa Postal 131, Campus Samambaia, Goiânia, 74001-970, Brazil.

[petrofez@uol.com.br](mailto:petrofez@uol.com.br)

**Table S1** Number of Fungal and Plant reads/contig and their length distribution obtained from suppression subtractive hybridization (SSH) derived from *Phaseolus vulgaris* L. during *Sclerotinia sclerotiorum* infection.

| Contig | Size (BP) | Accession number | Gene Description | *E* value | Total ESTs in Contig |
| --- | --- | --- | --- | --- | --- |
| ***Fungal*** | | | | | |
| Contig 1 - SS_001 | 553 | XP_390440.1 | glutamine synthetase (*Gibberella zeae*) | 8e-61 | 08 |
| Contig 3 - SS_003 | 322 | XP_001593397.1 | perilipin mpl1-like protein (*Metarhizium flavoviride var minus*) | 5e-29 | 04 |
| Contig 5 - SS_004 | 549 | XP_001593397.1 | perilipin mpl1-like protein (*Metarhizium flavoviride var minus*) | 2e-28 | 07 |
| Contig 6 - SS_005 | 229 | XP_003717922.1 | homoserine dehydrogenase (*Magnaporthe oryzae*) | 2e-11 | 06 |
| Contig 8 - SS_006 | 861 | NP_826813.1 | endochitinase (*Streptomyces avermitilis*) | 2e-82 | 02 |
| Contig 9 – SS_007 | 321 | XP_001596270.1 | den domain-containing protein (*Sclerotinia sclerotiorum* 1980) | 2e-31 | 02 |
| Contig 10 - SS_008 | 465 | [ADH51542.1](http://www.ncbi.nlm.nih.gov/protein/296802087?report=genbank&log$=prottop&blast_rank=1&RID=CK586WUH011) | perilipin MPL1-like protein CAP20 (*Sclerotinia sclerotiorum* 1980) | 1e-07 | 10 |
| Contig 12 - SS_010 | 417 | BAB69489.1 | guanine nucleotide-binding protein beta subunit (*Fusarium oxysporum*) | 2e-65 | 04 |
| Contig 13 - SS_011 | 658 | XP_008088069.1 | acid protease (*Sclerotinia sclerotiorum* 1980) | 8e-15 | 02 |
| Contig 14 - SS_012 | 299 | EFY98676.1 | sorting nexin-41 (*Metarhizium anisopliae*) | 7e-20 | 02 |
| Contig 16 - SS_014 | 378 | EFY94441.1 | aspartyl protease (*Sclerotinia sclerotiorum* 1980) | 1e-20 | 03 |
| Contig 17 - SS_015 | 331 | EJP70905.1 | transcription initiation protein (*Beauveria bassiana*) | 2e-54 | 11 |
| Contig 18 - SS_016 | 728 | EHK19451.1 | beta-1,6-glucanase putative (*Sclerotinia sclerotiorum* 1980) | 5e-18 | 02 |
| Contig 21 - SS_019 | 606 | EFY99484.1 | tripeptidyl peptidase precursor (*Metarhizium anisopliae*) | 1e-14 | 02 |
| Contig 22 - SS_020 | 348 | EFZ04249.1 | NAD transhydrogenase (*Metarhizium anisopliae*) | 1e-32 | 02 |
| Contig 23 - SS_021 | 355 | ELQ33320.1 | antiviral helicase ski2 (*Magnaporthe oryzae* Y34) | 4e-38 | 02 |
| Contig 25 - SS_022 | 646 | [CCT67208.1](http://www.ncbi.nlm.nih.gov/protein/517315241?report=genbank&log$=prottop&blast_rank=21&RID=2V5DGUK5015) | serine peptidase putative (*Sclerotinia sclerotiorum* 1980) | 3e-43 | 16 |
| Contig 26 - SS_023 | 530 | XP_001592294.1 | serine endopeptidase (*Sclerotinia sclerotiorum* 1980) | 2e-53 | 02 |
| Contig 27 - SS_024 | 249 | EGR50086.1 | beta-xylosidase (*Sclerotinia sclerotiorum* 1980) | 3e-15 | 03 |
| Contig 28 - SS_025 | 696 | AAP15044.1 | alkaline proteinase (*Trichoderma hamatum*) | 9e-144 | 07 |
| Contig 29 - SS_026 | 449 | EHK26726.1 | glycoside hydrolase family 17 partial (*Trichoderma virens*) | 1e-11 | 05 |
| Contig 31 - SS_027 | 252 | EFQ30677.1 | catalase hydroperoxidase (*Glomerella graminicola*) | 1e-31 | 02 |
| Contig 33 - SS_029 | 550 | EJP66560.1 | α-glucosidase putative (*Sclerotinia sclerotiorum* 1980) | 3e-40 | 02 |
| Contig 34 - SS_030 | 415 | AAP15044.1 | alkaline proteinase (*Trichoderma hamatum*) | 9e-33 | 03 |
| Contig 36 - SS_032 | 335 | XP_961711.1 | GTP-binding protein gtr1 (*Neurospora crassa)* | 6e-46 | 02 |
| Contig 39 - SS_035 | 382 | [XP_007825222.1](http://www.ncbi.nlm.nih.gov/protein/629732276?report=genbank&log$=prottop&blast_rank=17&RID=2V70RX25015) | oligopeptide transporter (*Metarhizium anisopliae*) | 3e-30 | 02 |
| Contig 40 - SS_036 | 382 | XP_963374.1 | protein sey1 (*Sclerotinia sclerotiorum 1980*) | 6e-42 | 09 |
| Contig 41 - SS_037 | 370 | CAE47978.1 | oleate delta-12 desaturase (*Aspergillus fumigatus*) | 4e-30 | 05 |
| Contig 42 - SS_038 | 520 | EHK45968.1 | glucan endo-1,3-β-glucosidase (*Sclerotinia sclerotiorum* 1980) | 3e-22 | 02 |
| Contig 43 - SS_039 | 541 | CCF38756.1 | glycosyl (*Colletotrichum higginsianum*) | 1e-51 | 03 |
| Contig 44 - SS_040 | 417 | EFQ30677.1 | peroxidase/ catalase (*Sclerotinia sclerotiorum* 1980) | 2e-29 | 02 |
| Contig 45 - SS_041 | 804 | XP_001591700.1 | beta-1,4-glucanase (*Sclerotinia sclerotiorum* 1980) | 6e-73 | 05 |
| Contig 46 - SS_042 | 229 | XP_001275643.1 | mitochondrial deoxynucleotide carrier (*Aspergillus clavatus*) | 1e-11 | 02 |
| Contig 47 - SS_043 | 373 | EFZ02693.1 | class e vacuolar protein-sorting machinery protein hse1 (*Metarhizium anisopliae*) | 8e-28 | 03 |
| Contig 48 - SS_044 | 430 | XP_001586791.1 | sec14 cytosolic factor SS1G_11820 (*Sclerotinia sclerotiorum*) | 1e-71 | 02 |
| Contig 52 - SS_047 | 371 | CCE27416.1 | bar domain-containing protein (*Metarhizium acridum*) | 2e-26 | 02 |
| Contig 53 - SS_048 | 318 | ELA31202.1 | oxidoreductase 2-nitropropane dioxygenase (*Sclerotinia sclerotiorum* 1980*s*) | 9e-19 | 05 |
| Contig 57 - SS_052 | 392 | AAL84695.1 | β-1,3-glucanase precursor (*Sclerotinia sclerotiorum* 1980) | 6e-05 | 02 |
| Contig 58 - SS_053 | 679 | [AAN62894.1](http://www.ncbi.nlm.nih.gov/protein/24571157?report=genbank&log$=prottop&blast_rank=12&RID=CGX8SRV201N) | cell wall protein (*Saccharomyces cerevisiae*) | 3e-13 | 10 |
| Contig 59 - SS_054 | 665 | ELA27998.1 | oligopeptide transporter (*Colletotrichum gloeosporioides*) | 1e-72 | 03 |
| Contig 60 - SS_055 | 562 | CCF44746.1 | woronin body major protein (*Colletotrichum higginsianum*) | 1e-61 | 02 |
| Contig 61 - SS_056 | 257 | EGR51636.1 | translocation protein sec62 (*Sclerotinia sclerotiorum 1980*) | 1e-22 | 03 |
| Contig 62 - SS_057 | 481 | XP_001586789.1 | vacuolar protease A SS1G_11818 (*Sclerotinia sclerotiorum 1980*) | 4e-53 | 06 |
| Contig 63 - SS_058 | 411 | EFY98807.1 | proteasome component pre6 (*Sclerotinia sclerotiorum 1980*) | 3e-47 | 03 |
| Contig 64 - SS_059 | 301 | EFY98401.1 | mfs multidrug (*Metarhizium anisopliae*) | 5e-47 | 02 |
| Contig 65 - SS_060 | 395 | EJP65591.1 | NADH-ubiquinone oxidoreductase (*Beauveria bassiana*) | 8e-71 | 02 |
| Contig 66 - SS_061 | 502 | EFY99256.1 | ATP synthase beta chain (*Metarhizium anisopliae*) | 3e-102 | 02 |
| Contig 67 - SS_062 | 422 | BAF37919.1 | acetaldehyde dehydrogenase (*Claviceps purpurea*) | 4e-68 | 02 |
| Contig 74 - SS_068 | 221 | SS1G_00477 | ubiquitin homeostasis protein lub1 – phospholipase (*Sclerotinia sclerotiorum 1980*) | 1e-12 | 05 |
| Contig 75 - SS_069 | 331 | SS1G_10538 | ceramidase (*Sclerotinia sclerotiorum* 1980) | 6e-20 | 08 |
| Contig 76 - SS_070 | 497 | EGR51699.1 | predicted protein (*Trichoderma reesei*) | 3e-35 | 02 |
| Contig 77 - SS_071 | 297 | SS1G_09620 | hypothetical protein – GTPase (*Sclerotinia sclerotiorum 1980)* | 2e-04 | 08 |
| Contig 78 - SS_072 | 186 | XP_001594760.1 | pyruvate kinase (*Sclerotinia sclerotiorum 1980)* | 4e-14 | 02 |
| Contig 82 - SS_076 | 337 | CCF45341.1 | 26s protease regulatory subunit 4 (*Colletotrichum higginsianum*) | 3e-23 | 02 |
| Contig 84 - SS_078 | 395 | CCF34677.1 | zz type zinc finger domain-containing protein (*Colletotrichum gloeosporioides*) (*Sclerotinia sclerotiorum 1980)* | 6e-10 | 03 |
| Contig 86 – SS_80 | 417 | SS1G_14466 | geranylgeranyl pyrophosphate synthetase (biosynthesis of secondary metabolites) | 5e-25 | 03 |
| Contig 87 - SS_081 | 329 | EGR44538.1 | conidial pigment polyketide synthase alb1 (*Trichoderma reesei*) | 2e-20 | 06 |
| Contig 88 - SS_082 | 588 | CCF46208.1 | cytochrome p450 alkane hydroxylase (*Sclerotinia sclerotiorum 1980)*) | 4e-29 | 03 |
| Contig 89 - SS_083 | 827 | KDB11413.1 | GTP binding protein (*Villosiclava virens*) | 2e-39 | 02 |
| Contig 90 - SS_084 | 496 | EFQ30677.1 | catalase/peroxidase (*Colletotichum sublineola*) | 4e-54 | 04 |
| Contig 91 - SS_085 | 305 | EJP69242.1 | ser thr protein phosphatase family (*Beauveria bassiana*) | 2e-30 | 02 |
| Contig 92 - SS_086 | 554 | EHK17607.1 | dynamin family protein (*Trichoderma viren*) | 2e-28 | 02 |
| Contig 97 - SS_090 | 307 | EJT81032.1 | ph-response regulator protein (*Sclerotinia sclerotiorum* 1980) | 2e-09 | 02 |
| Contig 98 - SS_091 | 540 | SS1G_07146.3 | cellobiohydrolase (*Sclerotinia sclerotiorum* 1980) | 2e-40 | 02 |
| Contig 99 - SS_092 | 740 | EHK49081.1 | glycoside hydrolase family 18 protein (*Trichoderma atroviride*) | 2e-119 | 02 |
| Contig 101 -SS_094 | 270 | EHK44088.1 | phosphoenolpyruvate carboxykinase (*Trichoderma atroviride*) | 7e-22 | 02 |
| Contig 102 - SS_095 | 383 | XP_001272491.1 | high affinity methionine permease (*Aspergillus clavatus*) | 1e-31 | 02 |
| Contig 103 - SS_096 | 778 | XP_001588682.1 | GTP-binding protein (*Sclerotinia sclerotiorum* 1980) | 1e-51 | 02 |
| Contig 105 - SS_098 | 502 | EHK23397.1 | high-affinity glucose transporter (*Trichoderma virens*) | 1e-89 | 02 |
| Contig 106 - SS_099 | 221 | EJP67622.1 | homoserine dehydrogenase (*Beauveria bassiana*) | 3e-14 | 04 |
| Contig 108 - SS_101 | 743 | EGR45177.1 | glutaryl- dehydrogenase (*Trichoderma reese*) | 1e-80 | 02 |
| Contig 111 - SS_104 | 336 | XP_960423.1 | fructose- -bisphosphatase (*Neurospora crassa*) | 9e-30 | 02 |
| Contig 116 - SS_108 | 398 | EHK22417.1 | hypothetical protein TRIVIDRAFT (*Trichoderma virens*) | 3e-25 | 02 |
| Contig 117 - SS_109 | 380 | EGR50812.1 | glycoside hydrolase family 20 protein (*Trichoderma reesei*) | 1e-10 | 03 |
| Contig 118 - SS_110 | 580 | EFY98486.1 | glutamine synthetase (*Metarhizium anisopliae*) | 1e-48 | 03 |
| Contig 119 - SS_111 | 305 | XP_001210923.1 | mitochondrial phosphate carrier protein (*Aspergillus terreus*) | 1e-17 | 02 |
| Contig 120 - SS_112 | 390 | EJP65662.1 | cytochrome b-c1 complex subunit 2 (*Beauveria bassiana*) | 1e-61 | 02 |
| Contig 121 - SS_113 | 423 | EFY85620.1 | eukaryotic translation initiation factor subunit (*Metarhizium acridum*) | 6e-34 | 03 |
| Contig 123 - SS_115 | 446 | EHK50815 | glycoside hydrolase family 18 protein Chitinase III (*Trichoderma atroviride)* | 5e-23 | 04 |
| Contg 124 - SS_116 | 1032 | XP_001586359.1 | glycosyl hydrolase (*Sclerotinia sclerotiorum* 1980) | 2e-32 | 09 |
| Contig 125 - SS_117 | 749 | CCF36376.1 | mac1 interacting protein 1 (*Colletotrichum higginsianum*) | 2e-14 | 02 |
| Contig 126 - SS_118 | 461 | XP_001258000.1 | beta-mannosidase (*Neosartorya fischeri*) | 3e-33 | 02 |
| Contig 127 - SS_119 | 400 | EGR52289.1 | tyrosine-protein phosphatase (*Trichoderma reesei*) | 4e-27 | 02 |
| Contig 129 - SS_121 | 582 | [XP_001593152.1](http://www.ncbi.nlm.nih.gov/protein/156054452?report=genbank&log$=prottop&blast_rank=9&RID=CE8VCBWH01N) | hypothetical protein SS1G_06074 (*Sclerotinia sclerotiorum* 1980) | 2e-14 | 18 |
| Contig 131 - SS_123 | 331 | EFY85061.1 | cystathionine beta-synthase (*Metarhizium acridum*) | 1e-36 | 02 |
| Contig 132 - SS_124 | 751 | [XP_002484510.1](http://www.ncbi.nlm.nih.gov/protein/242805359?report=genbank&log$=prottop&blast_rank=1&RID=CGSP17EE01S) | hypothetical protein TSTA_040370 (*Talaromyces stipitatus*) | 2e-50 | 13 |
| Contig 135 - SS_127 | 655 | ELA27998.1 | oligopeptide transporter (*Colletotrichum gloeosporioides*) | 4e-74 | 07 |
| Contig 139 - SS_131 | 654 | EJP70027.1 | major facilitator superfamily transporter (*Beauveria bassiana*) | 5e-63 | 03 |
| Contig 142 – SS_133 | 336 | SS1G_00477 | Phospholipase (*Sclerotinia sclerotiorum* 1980) | 1e-12 | 05 |
| Contig 143 - SS_134 | 312 | EJP63000.1 | exocyst complex component sec10 (*Beauveria bassiana*) | 6e-40 | 02 |
| Contig 144 - SS_135 | 553 | CAA10978.1 | protein disulfide isomerase (*Trichoderma reesei)* | 8e-63 | 02 |
| Contig 145 - SS_136 | 431 | EHK25266.1 | beta-1,3-glucanase (*Sclerotinia sclerotiorum* 1980) | 1e-64 | 08 |
| Contig 148 - SS_139 | 303 | EFY92509.1 | sec24 related gene family (*Sclerotinia sclerotiorum* 1980) | 3e-31 | 06 |
| Contig 149 - |  |  | No significant similarity found |  | 13 |
| Contig 150 - SS_140 | 317 | XP_003652726.1 | glycoside hydrolase family 35 protein (*Thielavia terrestris*) | 1e-21 | 02 |
| Contig 153 - SS_143 | 569 | ELA26500.1 | 2-nitropropane dioxygenase (*Sclerotinia sclerotiorum* 1980) | 2e-44 | 02 |
| Contig 155 - SS_145 | 316 | EHK25869.1 | glycoside hydrolase family 2 protein (*Trichoderma virens*) | 2e-20 | 04 |
| Contig 158- SS_148 | 597 | EGX91377.1 | cytochrome p450 51b (*Cordyceps militari*) | 5e-111 | 02 |
| Contig 159 - SS_149 | 388 | EFY92417.1 | beta-1,3-glucan synthase catalytic subunit (*Metarhizium acridum*) | 6e-51 | 02 |
| Contig 160 - SS_150 | 290 | XP_001577682.1 | GTP-binding protein (*Sclerotinia sclerotiorum* 1980) | 6e-33 | 04 |
| Contig 161 - SS_151 | 425 | ELA31513.1 | zinc transcription factor (*Sclerotinia sclerotiorum* 1980) | 2e-44 | 28 |
| Contig 163 - SS_153 | 221 | ELA23385.1 | polysaccharide deacetylase family protein (*Colletotrichum gloeosporioides*) | 6e-22 | 03 |
| ***Plant*** | | | | | |
| Contig 04 - SSPV_004 | 635 | CAA09212.1 | RNA helicase [*Arabidopsis thaliana*] | 2e-34 | 3 |
| Contig 02 - SSPV_002 | 489 | AAF19401.1 | phosphoenolpyruvate carboxylase kinase [*Glycine max*] | 3e-72 | 13 |
| Contig 03 - SSPV_003 | 607 | AAD34548.1 | cystathionine gamma-synthase [*Glycine max*] | 1e-14 | 5 |
| Contig 5 - SSPV_045 | 568 | XP_003520228.1 | ferredoxin-nadp reductase [*Glycine max*] | 2e-16 | 3 |
| Contig 06 - SSPV_006 | 657 | AT3G20820 | leucine rich repeat protein [*Arabidopsis thaliana*] | 4e-47 | 5 |
| Contig 07 - SSPV_007 | 489 | XP_006601343.1 | auxin response factor-like protein [*Glycine max*] | 2e-57 | 7 |
| Contig 08 - SSPV_008 | 653 | CAA47345.1 | heat shock protein 70 [*Phaseolus vulgaris*] | 1e-94 | 3 |
| Contig 09 - SSPV_009 | 556 | AAX31514.1 | DNA mismatch repair protein [*Phaseolus vulgaris*] | 2e-144 | 4 |
| Contig 10 - SSPV_010 | 488 | ATCG01130 | chloroplast chlorophyll a b-binding protein [*Arabidopsis thaliana*] | 8e-30 | 3 |
| Contig 11 - SSPV_011 | 532 | AT4G35770 | senescence-associated protein [*Arabidopsis thaliana*] | 4e-60 | 4 |
| Contig 12 - SSPV_012 | 483 | AET79244.1 | diphenol oxidase laccase [*Glycine max*] | 5e-63 | 3 |
| Contig 13 - SSPV_013 | 433 | AGV54475.1 | Lipoxygenase [*Phaseolus vulgaris*] | 5e-22 | 3 |
| Contig 14 - SSPV_014 | 501 | CAA59482.1 | pectin methylesterase [*Phaseolus vulgaris*] | 1e-58 | 3 |
| Contig 15 - SSPV_015 | 538 | BAI68016.**1** | ATP synthase f1 subunit 1 [*O. sativa*] | 5 e-70 | 3 |
| Contig 16 - SSPV_016 | 665 | XP_006583980.1 | S-adenosylmethionine synthetase [*Glycine max*] | 4 e-79 | 3 |
| Contig 17 - SSPV_017 | 590 | NP_177796 | DNAJ heat shock protein [*Arabidopsis thaliana*] | 1e-112 | 5 |
| Contig 18 - SSPV_018 | 325 | AGV54715.1 | xyloglucan endotransglucosylase hydrolase [*Phaseolus vulgaris*] | 4e-102 | 3 |
| Contig 19 - SSPV_019 | 559 | AAP69867 | glutathione peroxidase 1 [*Lotus japonicus*] | 1e-32 | 3 |
| Contig 20 - SSPV_020 | 457 | CAB16852 | beta-galactosidase like protein [*Arabidopsis thaliana*] | 2e-18 | 4 |
| Contig 21 - SSPV_021 | 308 | P06215 | Endochitinase precursor [*Phaseolus vulgaris*] | 3e-75 | 3 |
| Contig 22 - SSPV_022 | 502 | AAK84883 | NAC domain protein NAC1 [*Phaseolus vulgaris*] | 1e-23 | 3 |
| Contig 23 - SSPV_023 | 555 | XP_003526970.1 | aspartyl aminopeptidase-like [*Gycine max*] | 1e-56 | 6 |
| Contig 24 - SSPV_024 | 689 | AES79588.2 | proteasome subunit alpha [*Medicago truncatula*] | 9 e-54 | 9 |
| Contig 25 - SSPV_025 | 368 | AHA84124.1 | elongation factor 1 [*Phaseolus vulgaris*] | 7 e-37 | 6 |
| Contig 26 - SSPV_026 | 601 | P06215 | class I chitinase [*Phaseolus vulgaris*] | 1e-17 | 3 |
| Contig 27 - SSPV_027 | 650 | AAQ20042 | cytochrome P450 [*Medicago truncatula*] | 1 e-88 | 3 |
| Contig 28 - SSPV_028 | 509 | XP_003528630.1 | polygalacturonase-like protein [*Gycine max*] | 8e-14 | 3 |
| Contig 29 - SSPV_029 | 336 | AAR26001 | endo-1,3-beta-glucanase [*Glycine max*] | 1e-34 | 3 |
| Contig 30 - SSPV_030 | 439 | AAB00098 | G-box binding factor [*Gycine max*] | 2e-29 | 3 |
| Contig 31 - SSPV_031 | 651 | AAZ79659 | sucrose synthase [Vigna angularis] | 1e-28 | 2 |
| Contig 32 - SSPV_032 | 408 | AAB97165 | ribulose 1,5-bisphosphate carboxylase/oxygenase small subunit [*Phaseolus vulgaris*] | 2e-31 | 10 |
| Contig 33 - SSPV_033 | 532 | AAZ79659 | Putative cellulose synthase [*Fagus sylvatica*] | 1e-10 | 3 |
| Contig 34 - SSPV_034 | 568 | AAA50172 | Chlorophyll a/b-binding protein [*Glycine max*] | 5e-19 | 3 |
| Contig 35 - SSPV_035 | 640 | AFF57838.1 | Peroxidase [*Phaseolus vulgaris*] | 9e-57 | 2 |
| Contig 36 - SSPV_036 | 441 | XP_003555942.1 | ribose 5-phosphate isomerase [*Glycine max*] | 4e-21 | 3 |
| Contig 37 - SSPV_037 | 611 | AEA47059.1 | proteasome subunit alpha (*M. tuncatula)* | 1e-42 | 2 |
| Contig 38 - SSPV_038 | 341 | AAC37357.1 | Catalase [*Zea mays*] | 7e-69 | 3 |
| Contig 40 - SSPV_040 | 489 | AGZ15378.1 | fructose bisphosphate aldolase [*Phaseolus vulgaris*] | 2e-123 | 3 |
| Contig 41 - SSPV_041 | 621 | AGV54395.1 | s-adenosylmethionine decarboxylase [*Phaseolus vulgaris*] | 2e-126 | 3 |
| Contig 42 - SSPV_042 | 406 | CAA40339.1 | ribulose- -bisphosphate carboxylase oxygenase small subunit [*Phaseolus vulgaris*] | 3e-52 | 5 |
| Contig 43 - SSPV_043 | 431 | AEE32999.1 | 26s protease regulatory subunit [*Arabidopsis thaliana*] | 3e-42 | 3 |
| Contig 44 - SSPV_044 | 549 | CAB52473.1 | ATP synthase beta subunit [*Arabidopsis thaliana*] | 3e-23 | 5 |
| Contig 45 - SSPV_045 | 453 | AEE34128.1 | glutamine amidotransferase subunit [*Glycine max*] | 4e-37 | 2 |
| Contig 46 - SSPV_046 | 307 | AAC78474.1 | GDP-mannose pyrophosphorylase [*Arabidopsis thaliana*] | 1e-31 | 2 |
| Contig 48 - SSPV_048 | 455 | NP_001237049.1 | ferritin- chloroplastic [*Glycine max*] | 1e-47 | 2 |
| Contig 49 - SSPV_049 | 297 | ACJ61476.1 | ubiquitin-protein ligase [*Glycine max*] | 1e-59 | 5 |
| Contig 50 - SSPV_050 | 346 | AAD33696 | PR1 a precursor [*Glycine max*] | 1e-63 | 3 |
| Contig 51 - SSPV_051 | 289 | XP_007152897 | hypothetical protein/ kinase family protein [*Phaseolus vulgaris*] | 3e-60 | 3 |
